# A Dual Read-Out Assay to Evaluate the Potency of Compounds Active against *Mycobacterium tuberculosis*

**DOI:** 10.1371/journal.pone.0060531

**Published:** 2013-04-04

**Authors:** Juliane Ollinger, Mai Ann Bailey, Garrett C. Moraski, Allen Casey, Stephanie Florio, Torey Alling, Marvin J. Miller, Tanya Parish

**Affiliations:** 1 Infectious Disease Research Institute, Seattle, Washington, United States of America; 2 Department of Chemistry and Biochemistry, University of Notre Dame, Notre Dame, Indiana, United States of America; Johns Hopkins University School of Medicine, United States of America

## Abstract

Tuberculosis is a serious global health problem caused by the bacterium *Mycobacterium tuberculosis*. There is an urgent need for discovery and development of new treatments, but this can only be accomplished through rapid and reproducible *M. tuberculosis* assays designed to identify potent inhibitors. We developed an automated 96-well assay utilizing a recombinant strain of *M. tuberculosis* expressing a far-red fluorescent reporter to determine the activity of novel compounds; this allowed us to measure growth by monitoring both optical density and fluorescence. We determined that optical density and fluorescence were correlated with cell number during logarithmic phase growth. Fluorescence was stably maintained without antibiotic selection over 5 days, during which time cells remained actively growing. We optimized parameters for the assay, with the final format being 5 days’ growth in 96-well plates in the presence of 2% w/v DMSO. We confirmed reproducibility using rifampicin and other antibiotics. The dual detection method allows for a reproducible calculation of the minimum inhibitory concentration (MIC), at the same time detecting artefacts such as fluorescence quenching or compound precipitation. We used our assay to confirm anti-tubercular activity and establish the structure activity relationship (SAR) around the imidazo[1,2-*a*]pyridine-3-carboxamides, a promising series of *M. tuberculosis* inhibitors.

## Introduction

More than one third of the world’s population is infected with *Mycobacterium tuberculosis* and nearly 1.5 million people died from tuberculosis in 2010 [1]. A multidrug regimen taken for at least 6 months was established in the 1970s and is still used today. However, the high burden of tuberculosis infections in regions with limited health care resources has led to frequent treatment interruption and subsequent failure resulting in the rise of multi-drug resistant (MDR), and extensively drug resistant (XDR) strains. Recently, totally drug resistant (TDR) strains of *M. tuberculosis* have been isolated highlighting the urgency for the development of new treatments [2]. A number of drug candidates with anti-tuberculosis activity are currently in pre-clinical and clinical development [3] [4] [5]. However, many of these drug candidates are derivatives of current anti-tubercular drugs or they target the same cellular process and are therefore likely to help only with the treatment of drug sensitive *M. tuberculosis* infections [6] [7]. To be able to successfully tackle the problem of drug resistant *M. tuberculosis* we need novel compounds that target novel biological pathways in *M. tuberculosis,* shorten therapy, and reduce the burden of latent infection [8].

There has been an increasing focus on identifying new anti-tubercular agents from screening campaigns, either against single targets in biochemical assays [9] [10] [11] or against live organisms in whole cell screens [12] [13] [14]. Both activities lead to large numbers of compounds for which follow-up confirmatory activity is required; the most common route is the determination of the minimum inhibitory concentration (MIC) against actively growing *M. tuberculosis*. Traditionally MICs have been pursued using low throughput methods such as the serial proportion dilution method on agar plates [15] or spectrophotometric methods [16].

Assays with higher capacity have been developed and optimized for the use in *M. tuberculosis*, such as luciferase-based assays [17] [18], and Alamar Blue or tetrazolium assays [19] [20]. Assays using redox dyes or firefly luciferase are available in 96-well format, but the necessary addition of reagents during the assay is a major disadvantage since it increases the risk of contamination, and, more importantly for automation, increases the complexity of the assay. Use of the *lux* operon abolishes the need for an exogenous substrate, but there are no data on the reproducibility or robustness of the assay [18] [21]. Assays using *M. tuberculosis* expressing green fluorescent protein (Gfp) [22] [23] or using simple spectrophotometric analysis at 600 nm have also been developed [24]. These assays allow direct detection of *M. tuberculosis* growth and circumvent the drawbacks of the dye-based methods. However, these methods rely on a single correlate of growth and are subject to interference or artefacts of the assay. For example fluorescence quenching could lead to false positives, or compound precipitation could mimic growth and lead to false negatives. To assess the activity of new compounds reproducibly we focused on developing an assay with a dual readout of growth in a 96-well format that was amenable to automated liquid dispensing of compounds and bacterial culture. Combining fluorescence and an optical density based readout provided additional internal control and eliminated false positive or negative results.

We generated a strain of *M. tuberculosis* (CHEAM3) expressing a codon-optimized mCherry fluorescent protein (TOP_red_); this reporter is stably expressed in *M. tuberculosis* under a variety of conditions and its expression does not affect growth or virulence [25]. Using this strain allowed us to develop an assay that is amenable to 96-well format, and is robust and reproducible. Inhibition values for the two readouts were almost identical allowing for quick identification of assay artefacts (seen as discordance between the two MIC values). Work with similar recombinant strains has confirmed that such strains retain full virulence and have no fitness cost compared to the wild-type strain [25]. The disadvantage of this method is the need to construct a recombinant strain for each method, although this is also the case for luciferase-based assays (only OD and redox dyes can be used for non-recombinant strains). For non-recombinant strains, a single readout of OD can be used.

The imidazo[1,2-*a*]pyridine class of compounds shows promise as a synthetically accessible and potent *in vitro* anti-tubercular compound class [26], [27], [28]. We tested twenty imidazo[1,2-*a*]pyridine compounds in our 96-well MIC assay. Our results show that these compounds are potent inhibitors of *M. tuberculosis* growth and a promising starting point for the development of a novel anti-tuberculosis treatment.

## Materials and Methods

### Culture of *M. tuberculosis*

*M. tuberculosis* H37Rv was grown in Middlebook 7H9 medium supplemented with 0.05% w/v Tween 80, 10% v/v oleic acid albumin dextrose catalase (OADC) supplement (Becton Dickinson) (7H9-Tw-OADC) or on Middlebrook 7H10 agar containing 10% v/v OADC. Hygromycin was added to 50 µg/mL where required (7H9-Tw-OADC-H). Small scale cultures were grown in 10 mL medium in 50 mL conical tubes; large scale cultures were grown in 100 mL medium in 450 cm^2^ roller bottles. Recombinant *M. tuberculosis* expressing codon-optimized mCherry (TOP_red_) under the control of a strong, constitutively expressed promoter, was generated by electroporating the pCherry3 plasmid [25] into *M. tuberculosis* H37Rv (London Pride) [29] and isolating transformants on plates containing hygromycin.

Frozen seed stocks were prepared as follows: 10 mL 7H9-Tw-OADC-H was inoculated with a cell stock, grown to logarithmic phase (OD_590_ = 0.4–0.9) and used to inoculate 100 mL of 7H9-Tw-OADC-H in a 450 cm^2^ roller bottle to a theoretical OD_590_ of 0.01. The large scale culture was incubated rolling at 37°C until OD_590_ = 1.0. Aliquots of 1 mL of this culture were made in 2 mL cryovials and stored at −80^ò^C for up to 6 months.

### Growth Curves for *M. tuberculosis* in 96-well Plates

*M. tuberculosis* was cultured in 96-well plates at 37°C static as follows: 50 µL of 7H9-Tw-OADC, 4% DMSO medium was added to columns 1–12 of 96-well black, clear bottom plates (Greiner) using a matrix electronic multichannel pipette. *M. tuberculosis* was grown in large scale culture; 30 mL of cells were harvested, filtered through a 5 µm filter and adjusted to an OD_590_ of 0.03, 0.04 or 0.05. 10 mL of the culture was transferred into a sterile trough and 50 µL culture added to columns 1–11 of the 96-well plates using a multi-channel pipette. Plates were incubated in sealed bags in a humidified incubator at 37°C. Fluorescence and OD_590_ were measured on days 0, 3, 4, 5 and 6; a single plate was used for each time point. To generate the DMSO dose response curves, serial dilutions of DMSO starting at 40% DMSO were prepared in 7H9-Tw-OADC in 96-well plates. Plates were read after 5 days and % growth was calculated as compared to 7H9-Tw-OADC (no DMSO). Colony forming units (CFU) were counted by plating serial dilutions on Middlebrook 7H10 solid agar medium plus 10% v/v OADC and incubating for 3–4 weeks at 37°C.

### Compound Preparation

Imidazo[1,2-*a*]pyridine compounds were synthesized as described [27] [26] and were of >95% purity determined as by ^1^H NMR, HPLC and HRMS analysis with exception of compound **13** which was purchased from Sigma. Streptomycin, isoniazid, ethambutol ofloxacin, ethambutol, ethionamide, and rifampicin were purchased from Sigma. Solid compounds were stored at RT in a low humidity environment in the dark. Compounds were solubilized in 100% DMSO to a concentration of 10 mM; 50 µL aliquots were prepared in 1.4 mL v-bottom matrix tubes and stored at −20°C. Compound aliquots were thawed at RT, diluted to 1 mM in 100% DMSO and shaken at 100 rpm overnight before use.

### Preparation of Compound Plates for Final Assay

Sterile 96-well, black-clear bottom plates (Greiner) were barcoded using a Zebra 90xiII printer and the BarTender Label Printing Software, version 8.01. Medium and compounds were dispensed into plates using a Biomek 3000 automated liquid handling workstation and a 8 channel 200 µL handling tool (MP200) contained in a custom laminar HEPA enclosure with an automatic pneumatic access port.

Each plate was prepared as follows: (i) 50 µL of 4 µM rifampicin in 7H9-Tw-OADC (2X maximum inhibition control) was dispensed into rows 1–4 of column 1; (ii) 50 µL of 4% DMSO in 7H9-Tw-OADC (2X minimum inhibition control) was dispensed into rows 5–8 of column 1; (iii) 96 µL of 7H9-Tw-OADC was dispensed into column 2; (iv) 50 µL of 4% DMSO +7H9-Tw-OADC was dispensed into columns 3–11; (v) 100 µL of 2% DMSO in 7H9-Tw-OADC (contamination control) was dispensed into column 12. Serial dilutions of compounds were prepared as follows: 4 µL of 1 mM compound stock was added to column 2 followed by a mix step and subsequent 10-step 2-fold dilution across the plate (column 2–11); tips were changed between each dilution step.

**Figure 1:**
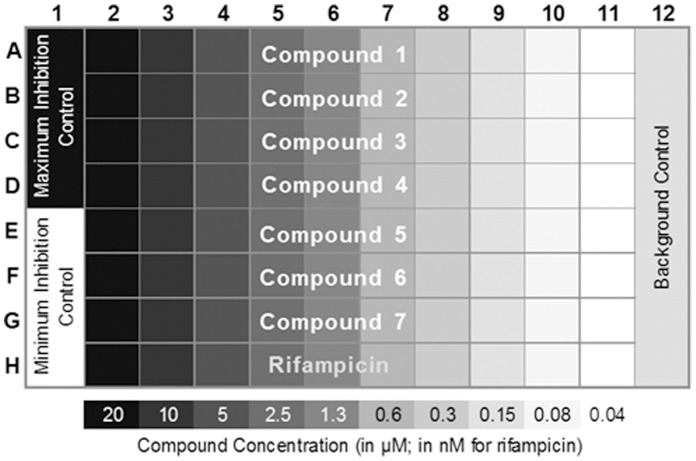
Final assay plate layout. The plate format is shown. Column 1 contains maximum inhibition control (rifampicin) in Rows A–D and minimum inhibition control (no compound) in Rows E–H. Column 12 is not inoculated with cells. Compounds are tested across each Row A–G in a two-fold serial dilution starting at 20 µM – concentrations are indicated below. A rifampicin dilution series is included in Row H starting at 20 nM.

For the final assay a standard plate layout included maximum inhibition control wells (2 µM rifampicin in 7H9-Tw-OADC), minimum inhibition control wells (2% DMSO in 7H9-Tw-OADC,), contamination control wells (2% DMSO in 7H9-Tw-OADC; not inoculated), and a 10 point rifampicin dose response curve starting at 20 nM (Figure 1).

### Inoculation of Assay Plates with *M. tuberculosis*

A 100 mL large scale culture was incubated rolling for 4 d; the OD_590_ was adjusted to 0.2 with 7H9-Tw-OADC-H and the culture was incubated for an additional 2 d to reach an OD_590_ of 0.8–0.9. 40 mL *M. tuberculosis* culture was harvested and filtered through a 5 µm syringe cellulose-acetate membrane filter using a 60 mL syringe to remove large clumps.

The filtered culture was adjusted to an OD_590_ of 0.04 using 7H9-Tw-OADC in a large mouth dispensing vessel; 300 mL was prepared for each run. A Multidrop Combi reagent dispenser (Thermo Scientific) fitted with a standard 8 channel cassette was used to inoculate the assay plates as follows: the cassette tubing was submerged in the cell suspension; the tubing was primed by dispensing 100 µL of culture into each well of a single plate (the prime plate was discarded); the assay plates were inoculated with 50 µL cell suspension per well except for column 12 (contamination/background control). Plates were lidded, placed into individual sealed bags, and incubated in a humidified water-jacketed incubator at 37°C for 5 days to prevent evaporation and edge effects. The position of each assay plate was recorded. Contamination checks were carried out at each stage by plating 100 µL of cells onto LB agar plates and checking for lack of growth after 24 h incubation at 37°C.

### Signal Detection and Data Analysis

Assay plates were removed from the sealed bags and the lids discarded. Each plate was sealed using silicon-adhesive sealing film (Excel Scientific), placed into a sealed bag and shaken for 5 min. Plates were removed from the bags for growth measurement at 37°C. Fluorescence was measured using a Synergy 4 plate reader (Biotech) using Ex586nm/Em614nm wavelengths for the TOP_red_ fluorescent reporter. OD_590_ was measured using monochromator-based optics.

### Data Analysis

Growth inhibition was calculated separately for fluorescence and OD_590_. The average background value was calculated from the contamination control wells (medium-only) and subtracted from all other wells in columns 1–11. The average OD_590_ or fluorescence reading of the minimum inhibition controls (DMSO-only; 100% growth) was used to calculate % growth for each test well. A 10 point dose response curve for each compound was plotted as % growth and was fitted to the Gompertz model using GraphPad Prism 5. MICs were calculated from the inflection point of the fitted curve to the lower asymptote (zero growth) as described in [30].

### Quality Control

Accuracy and performance of the Biomek liquid handler and the Multidrop Combi dispenser was tested using 7H9-Tw-OADC containing 0.2% yellow dye. Plate uniformity was assessed from the mean, standard deviation and coefficient of variation (CV).

## Results and Discussion

### Fluorescence as a Correlate of Growth in *M. tuberculosis*

**Figure 2:**
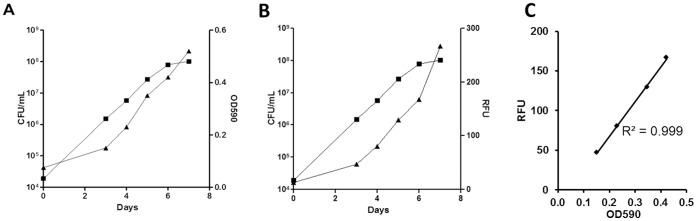
Correlation of fluorescence and optical density as measures of growth. A. Growth of *M. tuberculosis* CHEAM3 measured in 96-well plates. CFU – squares; OD_590_ - triangles. Cells were grown in 100 µL of 7H9-Tw-OADC in 96-well plates at a theoretical starting OD_590_ of 0.02. Results for OD_590_ are the average of all wells in the plate +/− SD. Samples were taken from 3 wells for CFU counts; results are the average +/− SD**. B.** Correlation of OD_590_ and RFU during growth in 96-well plates. Measurements were taken on days 3, 4, 5 and 6.

OD is often used as a surrogate of cell growth since the signal correlates with cell numbers. We confirmed that this was the case using our strain of *M. tuberculosis* under the conditions of the assay. We determined viable cell numbers (by measuring CFU) during growth in 96-well plates over 7 d. The viable cell count increased logarithmically until day 6, and this correlated with OD_590_ and fluorescence (Figure 2). By day 6 cells were entering stationary phase, at which point the CFU count remained steady. However, the OD_590_ and fluorescence continued to increase, which is likely due to the fact that spectrophotometric measurements cannot distinguish between viable and non-viable cells, nor between increases in cell size and/or cell wall thickness. Similarly the continuing increase in the fluorescence signal continued is likely due to the fact that expression is constitutive and remains active throughout stationary phase.

We tested the correlation between OD_590_ and fluorescence over time (Figure 2B). There was a linear correlation between the two readouts over a period of 6d. Therefore we concluded that both fluorescence and optical density could be used to follow the growth of the bacterial population. The correlation between CFU and fluorescence was similarly robust (Figure 2C).

### *M. tuberculosis* Maintains Logarithmic Growth Over 5 Days in 96-well Plates

We determined the minimum growth time needed for acceptable signal:background (>5) and signal:noise ratios (>10) for both fluorescence and OD. Selecting conditions that meet these minimal acceptable ratios balances the need for a sufficiently robust assay [31], while taking into account the slow growth rate of *M. tuberculosis*. The reporter strain was inoculated into medium and grown over 7 d in 96-well plates; OD and fluorescence were measured. As expected fluorescence was more sensitive with signal:background reaching 7 as early as day 4. The minimum growth time required to meet these criteria for both OD and fluorescence was 5 d (Table 1). The signal:background ratio of 6 for OD_590_, and 15 for fluorescence at day 6 compares favourably to the signal:background ratios reported by Franzblau, et. al. [32]on day 7 measured in the microplate Alamar blue assay (MABA). The inclusion of Tween 80 in our formulation likely contributes to the slightly lower signal:background ratio of our absorbance measurements. However, the signal:background ratio is higher for fluorescence measurements and is equivalent to those reported in Franzblau, et. al. [32] on day 7.

**Table 1:** Signal/background ratios for OD and RFU readouts.

	Signal/Background on Day			
	3	4	5	6
OD_590_	2	3	5	6
RFU	4	7	12	15

*M. tuberculosis* was grown in 96-well plates for the indicated length of time. Measurements were taken on days 3, 4, 5 and 6. Signal to background ratio was calculated by dividing the RFU or OD_590_ measurements from 80 test wells by the average signal in the background control wells containing 7H9-Tw-OADC plus 2% DMSO.

Our data demonstrated that the bacteria grow logarithmically for up to 6 d in 96-well plates before entering stationary phase. We selected day 5 as the optimal assay length to ensure that cells were in logarithmic phase; since the cell population is most uniform in terms of chemical and physiological properties during logarithmic phase, this should generate more consistent results, as well as provide the best signal increase during growth.

### Fluorescence Stability in the Absence of Antibiotic Selection

**Figure 3:**
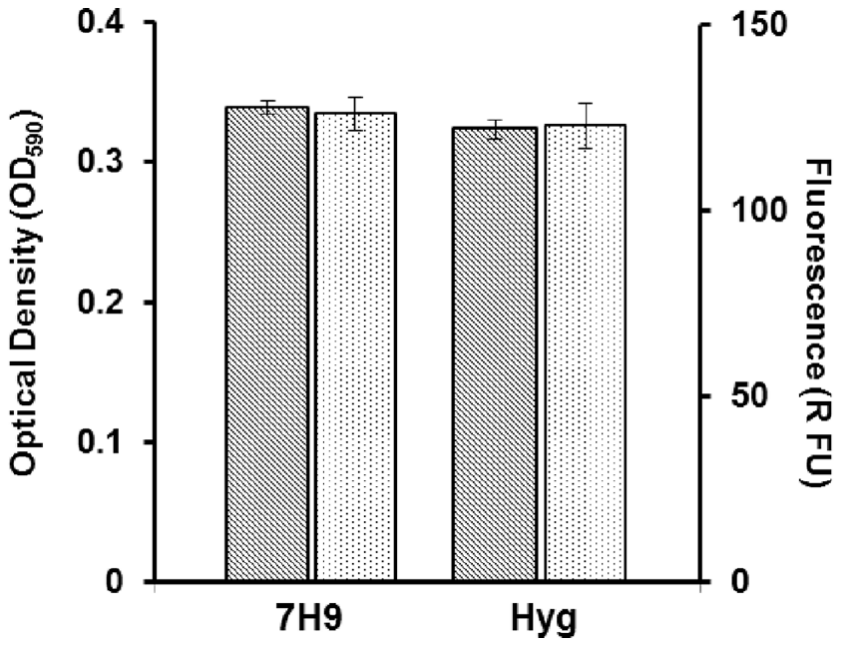
Stability of fluorescent signal in the absence of antibiotic selection. *M. tuberculosis* CHEAM3 was grown in 100 µL of 7H9-Tw-OADC-H or 100 µL of 7H9-Tw-OADC in 96-well plates. Fluorescence (dotted bars) and optical density (hashed bars) were measured after 5 d of incubation at 37°C. Measurements are average all of well. Results of 2 independent experiments are shown.

Once we had confirmed that fluorescence was a suitable correlate of growth, we determined whether antibiotic selection was required to maintain plasmid-mediated expression over a time period likely to be required for the assay. We tested the growth and fluorescence of the recombinant strain in 96-well plates in the presence and absence of hygromycin (Figure 3). The inclusion of hygromycin did not affect the endpoint of growth, and the absence of hygromycin did not affect the level of fluorescence after 5 d demonstrating that fluorescence is stable even without antibiotic selection. This confirmed that we would not require antibiotic selection during growth, thus avoiding any potential artefacts from interactions of test compounds with hygromycin.

### Optimization of Assay Parameters

We determined the optimum inoculum by varying the cell density. We tested inoculum densities using (theoretical) OD_590_ of 0.005, 0.02 and 0.04 corresponding to approximately 1×10^5^, 4×10^5^ and 8×10^5^ CFU/mL respectively. The optimum starting inoculum was an OD_590_ of 0.02 units (data not shown); using this inoculum cell density reached, but did not exceed, mid-logarithmic phase over 5 days (Figure 2). Higher starting densities resulted in cells entering late log to early stationary phase on Day 5 and lower theoretical starting densities resulted in inconsistent growth within the 5 day period (data not shown).

We investigated a number of other parameters to create a robust assay; these included optimizing the reader optics and testing multiple types of 96-well plates. The best conditions were those yielding the best signal:background (S:B), signal:noise (S:N), coefficient of variability (CV), day-to-day reproducibility and minimal plate-to-plate variability. Typically, the signal-to-background ratio was 5 for OD_590_ and 10 for RFU measurements. The signal-to-noise ratio exceeded 100 for both OD_590_ and RFU measurements. Intra-plate variability, measured by determining the coefficient of variation (%CV), was 3–4%. As expected for a biological cell-based assay, the largest variability in raw data was seen with biological replicates (inter-day variability). Because of this, positive, negative, and background controls were included on each plate. Rigorous validation and optimization of our assay condition resulted in the generation of highly reproducible MIC values. The average Z’ value of controls for 22 plates was 0.92±0.05 for fluorescence and 0.93±0.05 for OD readout with all plates scoring >0.70.

### The Effect of DMSO on *M. tuberculosis* Growth

**Figure 4:**
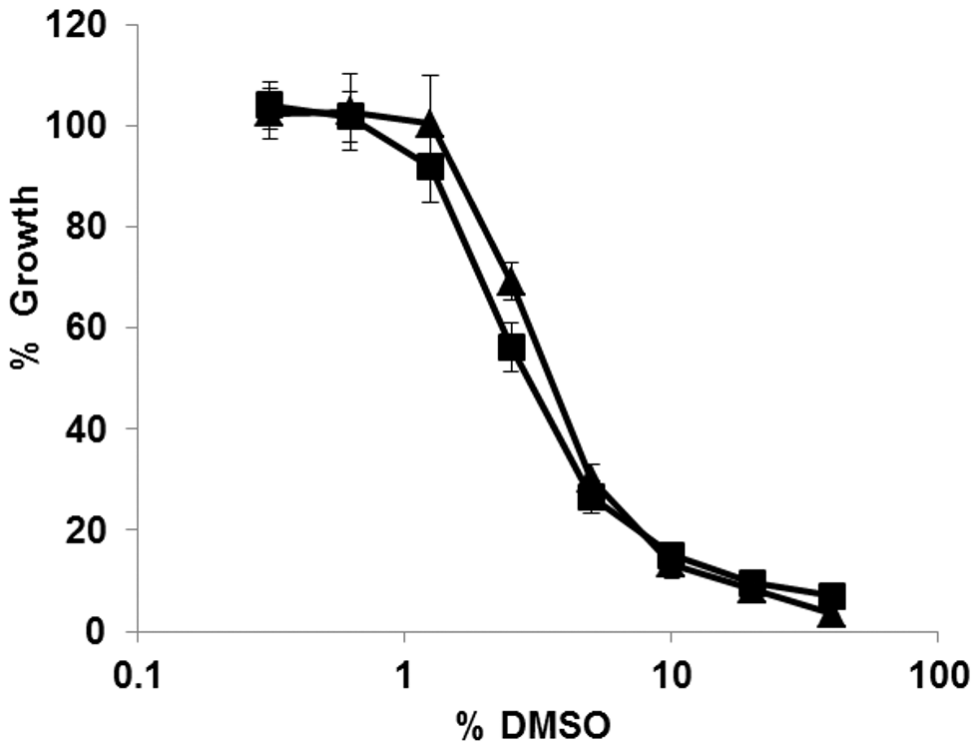
The effect of DMSO on growth of *M. tuberculosis*. *M. tuberculosis* growth was monitored in 96-well plates. DMSO was added as serial dilutions at a starting concentration of 40%. After 5 d of incubation at 37°C % growth was calculated by dividing RFU (triangles) or OD_590_ (squares) by the average of 4 control culture wells containing no DMSO. Results are the average of 8 independent dose response curves; error bars represent standard deviation.

Many compounds, particularly those used in drug discovery programs, have limited solubility in aqueous solution which can be improved by the addition of DMSO. We determined the maximum DMSO concentration that could be tolerated in our assay without a major effect on growth. A dose response curve was generated (Figure 4); 2.5% DMSO inhibited the growth of *M. tuberculosis* by 30–40%, which is unacceptably high, whereas 1.3% DMSO did not affect growth. Maintaining DMSO at or below 1.3% was not feasible as compound addition with the Biomek 3000 liquid handler was most consistent when transferring volumes ≥1.5 µL. Therefore we determined that our standard concentration of DMSO across the assay plate would be 2% to allow for a maximum flexibility during compound processing while minimizing the effect on bacterial growth to below 20% growth inhibition.

### Final Assay Conditions

The following final assays conditions were chosen to maximize assay robustness and reproducibility: 50 µL of 40 µM compound (in 4% DMSO, 7H9-Tw-OADC) plus 50 µL bacterial inoculum (in 7H9-Tw-OADC) at an OD of 0.04. Final assay volume is 100 µL; final DMSO concentration is 2%; final OD of culture is 0.02; incubation for 5 days at 37°C with humidification. Greiner black-clear bottom plates were chosen to maximize the signal-background ratio for both readouts and to minimize well-to-well crosstalk during the detection of fluorescence. Optical plate seals were chosen based on their adherence to the particular assay plates to ensure the biological containment of *M. tuberculosis*.

### Assay Reproducibility

**Figure 5:**
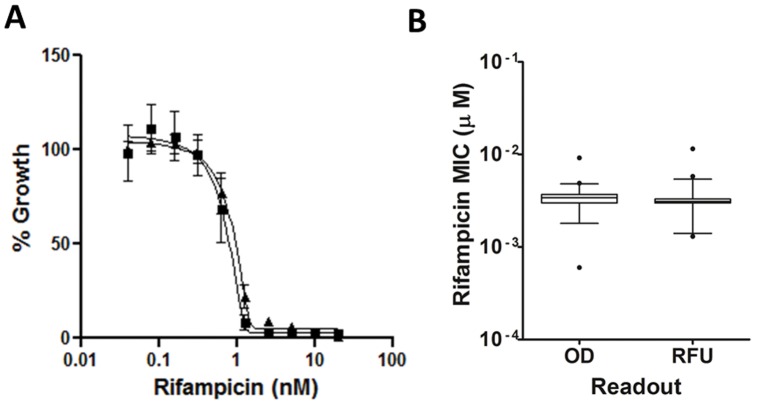
Assay reproducibility. **A** Dose response curve reproducibility for rifampicin. Results are the average of 8 independent dose response curves generated from the fluorescence +/− standard deviation. The MIC curves were generated using the Gompertz model **B** MIC reproducibility for rifampicin. MICs were calculated for rifampicin over a period of 12 months using fluorescence or OD. Results are for 337 independent rifampicin dose response curves are shown in a box and whiskers plot. MIC values that are outside the 99 percentile are indicated as outliers.

We determined the reproducibility of the assay using rifampicin as a control. We generated 337 individual rifampicin MICs, each run on a separate plate, over a period of 12 months. The mean rifampicin MIC was 3.4 nM +/− 0.6 nM using the OD_590_ readout and 3.3 +/− 1.3 nM using the RFU readout; thus, the values derived from OD and RFU were identical. Growth inhibition curves and MICs were consistent over time and between plates confirming a high degree of robustness in this assay (Figure 5).

Our rifampicin MIC values are 3–10 fold more potent than those reported by Franzblau *et al.* [32] in Middlebrook medium. This is likely due to the inclusion of Tween 80 in our medium, which is known to increase the efficacy of rifampicin [32]. In addition, the shorter time frame in our assay of 5 d, as compared to 7 d could lead to lower MICs as described for *Staphylococcus aureus* [30].

Franzblau *et al*. [32] also observed significant shifts in MIC dependent upon the readout method. We do not see this readout dependent shift in MICs. In fact, for most compounds tested in our laboratory, MIC values are remarkably similar independent of the readout used (data not shown). Since Tween 80 minimizes clumping of the bacteria in liquid culture, the inclusion of Tween 80 may results in a thorough exposure of the bacteria to compound and may neutralize interference in absorbance measurements due to clumping. These factors may therefore account for the equivalent MIC values obtained from the OD and RFU data. It is likely that a combination of all these factors contributes to the more potent values we observe for rifampicin. In general, we can conclude that our assay is sensitive, robust, and highly reproducible and that the use of a fluorescent reporter has allowed us to shorten the incubation time for MIC measurements in *M. tuberculosis.*

### Assay Validation Using Known Antibiotics

We determined the MIC of commonly used antibiotics to benchmark the performance of our assay and to compare across other studies; we selected anti-tubercular agents with reported potencies in the micromolar and nanomolar range (Table 2). Our results confirm that the MIC calculated using the OD_590_ readout correlates with the MIC calculated using the RFU readout. In addition our MIC values for isoniazid, ofloxacin, and ethambutol correlate well with those determined using other assays [23] [22]. For streptomycin, ethionamide and rifampicin we observed an approximate 10-fold difference of our reported MIC compared to that reported using a green-fluorescent reporter strain (GFPMA) or using the MABA assay. These differences are likely to be due to slight difference in the experimental procedure such as incubation period or the composition of the growth medium used.

**Table 2:** MIC of known anti-tuberculosis drugs.

Compound	MIC (µM)					
	OD_590_		RFU		(1)GFPMA	(1)MABA (visual)
Streptomycin	2±0.9		2±0.8		0.2–0.6	0.3
Isoniazid	0.2±0.07		0.3±0.07		0.16	0.16
Ofloxacin	0.7±0.02		0.8±0.005		1.1–2.2	2.2
Ethambutol	5±0.02		6±0.03		2.3–4.6	9.2–18
Ethionamide	6.6±0.4		8.1±1		0.93	0.93
Rifampicin	0.003±0.005		0.004±0.006		0.03–0.06	0.03

MICs were determined in liquid medium using both readouts of growth, OD and fluorescence (RFU). Results are the average and standard deviations of a minimum of two independent runs.

(1) MIC values as recorded by Changsen et al. [22].

### Screening of New Anti-tuberculosis Inhibitors

**Figure 6:**
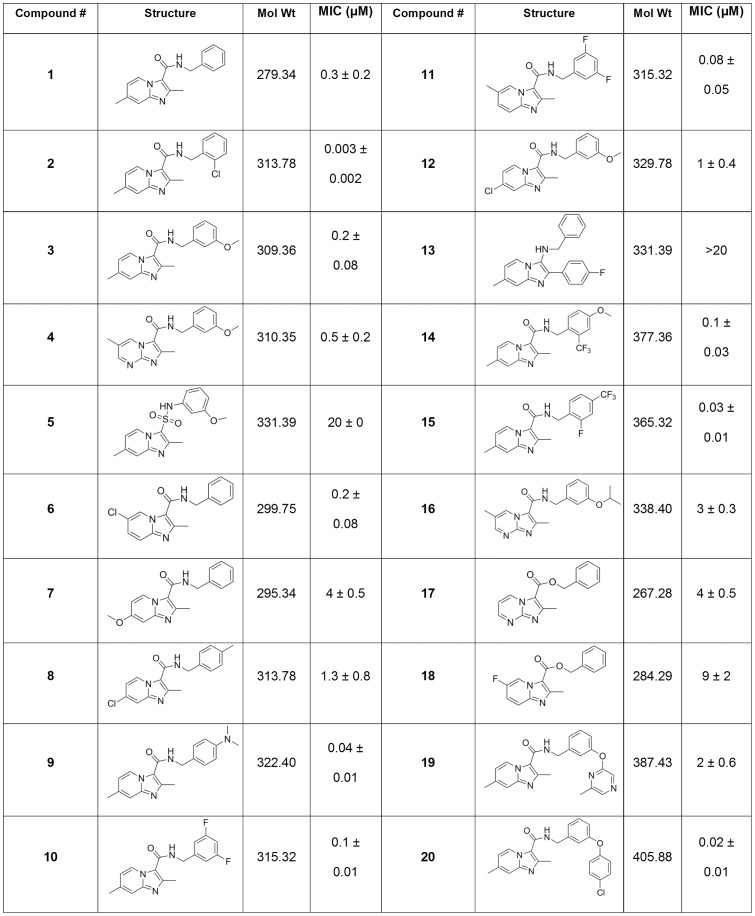
MIC determination of imidazo[1,2-a]pyridine compounds. MICs were determined in liquid medium using two readouts of growth (OD and fluorescence). The values from both readouts were combined and results are the average and standard deviations for a minimum of two independent runs.

Once we had confirmed that our assay was robust and reproducible, we tested a series of inhibitors [27]. This series was of interest since it showed good activity against *M. tuberculosis* and could be further developed. A diverse set of 20 imidazo[1,2-*a*]pyridines were tested using a standard plate layout; MICs were generally good and one compound was very potent, with an MIC lower than that of rifampicin (Figure 6, compound **2**). In general, this compound class had activity comparable to the range shown by the known TB drugs with the exception of compound **5** and **13** where MIC >20 µM (Figure 6). Again, the MIC for each compound was calculated using the OD and the RFU readout. The average difference in MIC between the two readouts was less than 1.5-fold providing an internal control.

These two compounds suggest some trends within the structure activity relationship (SAR) of this class as **5** has a 3-sulfonamide compared to the 3-amide moiety found on all the other compounds except for **13**. However, **13** has two structural changes with only a benzyl amine at the 3-position and a 4-fluorobenzyl at the 2-position where all the other compounds bear a 3-amide and a 2-methyl moiety. It is reasonable that either of these changes could account for the poor potency (>20 µM) of **5** or **13** compared to the other compounds. For instance, when compounds more similar to **13** lacking the 3-carboxyl group and having either a 2-aryl (or 2-pyridyl) were screened by the Molecular Libraries Screening Center Network (MLSCN), only seven of the twenty-seven imidazo[1,2-*a*]pyridines had MIC values ranging from 1.5 to 4.4 µM [33]. Interestingly, there was a 4-fold difference in potency between the isomeric compounds **10** and **11** where the compound with the methyl in the 6-postion (**11**) had better potency (MIC  = 0.03–0.04 µM) than the isomer with the methyl at the 7-position (**1**, MIC  = 0.13 µM). This trend of improved potency of the 6-methyl isomer was observed in other compounds subsequently screened (data not shown). When a nitrogen was added at the 8-position it appeared that the resulting imidazo[1,2-*a*]pyrimidines (compounds **5**, **16** and **17**) had decreased potency (up to 10 fold). Compounds **19** and **20** suggest that larger substituents like the bi-aryl ethers are also tolerated.

The potency of the previously published compounds (**1**–**4**) as well as rifampicin in this assay was lower than that previously determined using the MABA assay against H37Rv [26], [27]. This might be due to differences in inoculation density, the medium carbon source used (glucose or palmitate), or the incubation times. Additionally the MABA assay relies on a redox reaction of the Alamar blue dye which detects metabolic activity, rather than growth or viable cells as detected in our assay and they may not be strictly correlated. Notwithstanding, many of the imidazo[1,2-*a*]pyridines screened showed nanomolar potency against replicating *M. tuberculosis* with compound **2**, the N-(2-chlorobenzyl)-2,7-dimethylimidazo[1,2-*a*]pyridine-3-carboxamide, as the most potent, having single digit nanomolar potency.

### Conclusions

We have developed an automated method to determine the MIC of compounds against *M. tuberculosis* allowing a fast and reliable evaluation of compound potency in a 96-well format. We combined the fluorescent readout of a far-red codon optimized fluorescent reporter (TOP_red_) [25] with spectrophotometric analysis at 590 nm to measure the growth of *M. tuberculosis*. Both readouts were optimized to yield a precise and robust detection of *M. tuberculosis* growth in 96 well plates in the presence of test compounds as demonstrated by our extensive studies with rifampicin, standard anti-tubercular drugs and anew anti-tubercular class of imidazo[1,2-*a*]pyrimidines studies.

Using fluorescence to monitor bacterial growth allows sensitive detection of cell numbers, but auto-fluorescence of library compounds can be problematic [34]. OD can be problematic where compound insolubility and precipitation occurs. In both cases a false negative i.e. compounds would be recorded as being inactive, might occur. Combining the two measurements on the same samples allows rapid detection of assay artefacts, by simple fact of a lack of correlation between MICs between the two readouts.

We minimized assay variability using a Biomek 3000 liquid handling workstation for addition and dilution of test compounds and the MultiDrop dispenser for the inoculation of test wells with *M. tuberculosis*. Our results show that our automated assay using the combined fluorescent and spectrophotometric readout allows for a precise and robust MIC calculation.

We used our assay to determine the MIC of known anti tuberculosis drugs and showed that our results are comparable to those obtained with the previously described, GFP-based assay, MABA assay or the proportion dilution method [15], the gold standard when determining the MIC of novel compounds.
